# Developing evidence-based clinical practice guidelines in hospitals in Australia, Indonesia, Malaysia, the Philippines and Thailand: values, requirements and barriers

**DOI:** 10.1186/1472-6963-9-235

**Published:** 2009-12-15

**Authors:** Tari J Turner

**Affiliations:** 1 Monash Institute of Health Services Research, Monash University, Monash Medical Centre, Clayton, Victoria, Australia

## Abstract

**Background:**

Evidence-based clinical practice guidelines support clinical decision-making by making recommendations to guide clinical practice. These recommendations are developed by integrating the expertise of a multidisciplinary group of clinicians with the perspectives of consumers and the best available research evidence. However studies have raised concerns about the quality of guideline development, and particularly the link between research and recommendations. The reasons why guideline developers are not following the established development methods are not clear.

We aimed to explore the barriers to developing evidence-based guidelines in eleven hospitals in Australia, Indonesia, Malaysia, the Philippines and Thailand, so as to better understand how evidence-based guideline development could be facilitated in these settings. The research aimed to identify the value clinicians place on guidelines, what clinicians want in guidelines developed in hospital settings and what factors limit rigorous evidence-based guideline development in these settings.

**Methods:**

Semi-structured, face-to-face interviews were undertaken with senior and junior healthcare providers (nurses, midwives, doctors, allied health) from the maternal and neonatal services of the eleven participating hospitals. Interviews were audio-recorded, transcribed and a thematic analysis undertaken.

**Results:**

Ninety-three individual, 25 pair and eleven group interviews were conducted. Participants were clear that they want guidelines that are based on evidence and updated regularly. They were also clear that there are major barriers to this. Most of the barriers were shared across countries, and included lack of time, lack of skills in finding, appraising and interpreting evidence, lack of access to relevant evidence and difficulty arranging meetings and achieving consensus.

Barriers that were primarily identified in Australian hospitals include cumbersome organisational processes and a feeling that guidelines are being developed for bureaucratic ends. Barriers that were primarily identified in South East Asian hospitals include difficulty accessing evidence due to limited resources available for computers, internet and journal subscriptions and limited skills in computing and English.

**Conclusions:**

The clinicians in these eleven very different hospitals want evidence-based guidelines. However they are frustrated by guideline development processes that are enormously time, skill and resource intensive. They feel strongly that "there's got to be a better way".

The fact that the great majority of the identified barriers were shared across settings may provide an opportunity to develop a more pragmatic way of developing guidelines that can be applied in many contexts.

## Background

Evidence-based clinical practice guidelines (EBCPGs) support clinical decision-making by integrating the expertise of a multidisciplinary group of clinicians with the perspectives of consumers and the best available research evidence, to make recommendations to guide clinical practice.

As part of the move towards evidence-based health care, health organisations and clinician groups are increasingly required to develop and implement EBCPGs. There is some evidence to support the role of EBCPGs, with research demonstrating that in some situations, with rigorous development and thorough implementation, EBCPGs can improve both the quality and outcomes of care[1]

There are several guides to EBCPG development [2-7] and there is broad agreement among these guides on the key elements of the EBCPG development process[8] Despite this, EBCPGs are often not developed in a reliable way, and there is frequently very little link between the research included in EBCPGs and their recommendations [9-13]

Existing methods of EBCPG development have largely been produced at a national or international level, for use by organisations creating EBCPGs to guide widespread practice. The processes they describe often take years and hundreds of thousands of dollars to complete. This amount of time and money is not usually available in hospital settings, and so there are immediate significant barriers to EBCPG development at this level.

We undertook a needs analysis to explore the experience of guideline development and the enablers of and barriers to developing evidence-based guidelines in perinatal units in hospitals in Australia, Indonesia, Malaysia, the Philippines and Thailand, so as to better understand how evidence-based guideline development could be facilitated in these settings, and to explore how barriers to the development EBCPGs varied between settings.

## Methods

The research aimed to address three questions:

• What value do clinicians give evidence-based clinical practice guidelines in hospital settings?

• What do clinicians want in evidence-based clinical practice guidelines developed in a hospital setting?

• What factors limit rigorous evidence-based clinical practice guideline development in hospital settings?

To address these questions, we undertook a needs assessment using qualitative research methods, based on semi-structured, face-to-face interviews with clinicians from the maternal and neonatal services in two hospitals in each of Australia, Indonesia, Malaysia and the Philippines and three hospitals in Thailand. Both rural and metropolitan hospitals were included. The hospitals in South East Asia were participating in the SEA-ORCHID Project [14] (South East Asia - Optimising Reproductive and Child Health In Developing countries, http://www.seaorchid.org) which aimed to improve the quality of maternal and neonatal health care by increasing the generation, synthesis and use of health research. The existing low levels of evidence-based clinical practice guideline development and barriers to development specific to these hospitals have been reported previously [15,16]. The two Australian hospitals were included to facilitate investigation of the degree to which the barriers at the hospitals in South East Asia were shared beyond this region and to enable exploration of the experiences of staff at a large metropolitan tertiary teaching hospital as well as a smaller rural hospital.

At each hospital we aimed to interview two junior and two senior doctors, and two junior and two senior nurses or midwives, and appropriate allied health staff. Senior personnel working in the hospitals were asked to identify potential participants. Potential participants received a written explanatory statement and signed a consent form to indicate informed consent. Ethics approval was granted by Monash University and the appropriate ethics committees in the countries of the participating hospitals.

Interviews were loosely based on a pre-specified interview protocol (see Additional file 1), but the specific questions were varied according to the level of experience and previous responses of the interviewees. Participants were asked to describe the process for developing guidelines at their hospital, the barriers to development of evidence-based guidelines and how they thought guidelines should be developed. The interview protocol was piloted at two hospitals and then slightly revised for use in the other hospitals. In South East Asia the questions about guidelines formed part of a larger interview about evidence-based practice, in Australia the interviews focused specifically on guidelines.

Interpreters were used for the interviews where required. Interviews were audio recorded, de-identified and transcribed, and the data coded and then analysed in emerging themes using NVivo software. A subset of the data was independently coded by a second reviewer to confirm analysis.

## Results

Ninety-three individual, 25 pair and eleven group interviews were conducted. Fifty-three participants were interviewed in Thailand, 68 in Malaysia, 27 in Indonesia, 31 in the Philippines and 18 in Australia. Doctors and either nurses or midwives or both were interviewed at all sites. Allied health staff were only interviewed in Australia. All participating nurses/midwives and allied health staff were female. Both male and female doctors were interviewed at all sites. Male doctors represented approximately 30% of all doctors interviewed and were more common in senior positions and neonatal specialties. At each of the sites, the final interviews did not generate any new themes.

The interviews highlighted several themes including the value of and need for guidelines and the requirement for guidelines to be evidence-based, concise and clinically focused, credible, multidisciplinary and up-to-date. They also identified several barriers to development of guidelines, including lack of time, lack of skills, lack of access to evidence, difficulty arranging meetings and achieving consensus, and the role of adaptation of guidelines. A summary of the key points of these results is provided in Additional file 2.

Results are presented in these emerging themes with direct quotes (in italics) to illustrate concepts.

### The value of and need for guidelines

All participants agreed that there was a need for guidelines to support clinical practice. Guidelines were seen as important in providing guidance about best practice and supporting standardisation of practice. Participants thought guidelines were particularly useful for new or junior staff, and for infrequently performed practices.

*"I think that they're important and I think that we need them, because we need direction and we need consistency in our care." Senior Nurse, Australia, Female*.

However, there was a feeling among several participants from Australia that health services and clinicians were becoming over-reliant on guidelines or that guidelines were being seen as a panacea. Participants were concerned that guidelines were being developed as an unthinking reaction in clinical areas that were well understood rather than in areas of controversy or variation in practice where they would be more useful. Participants suggested that it might be more beneficial to focus on developing major guidelines in critical areas of clinical practice.

*"Because if something goes wrong once, there's an immediate thing 'Right, well we must have a guideline'. This reflex that we must have a guideline or we must change the guideline, I think is an inappropriate reflex." Senior Doctor, Australia, Female*.

While supportive of guidelines in principle, several senior Australian participants were very concerned that guidelines were increasingly being developed to meet bureaucratic or reporting requirements, rather than to support clinical practice and that, as a result, they were becoming *"notoriously useless" Senior Doctor, Australia, Male*.

These participants emphasised that if guidelines are to be useful they must be written by clinicians, to meet the needs of clinicians rather than administrators.

*"I've become more and more disenchanted with the preparation of clinical guidelines ... because I get the sense that clinical practice guidelines in my hospital are written for administrators and for key performance indicators for the [government]." Senior Doctor, Australia, Male*.

Some clinicians in each of the countries expressed doubts about whether guidelines were being used.

Q: "Do you think guidelines are useful?"

A: "It is useful ...if most of us read them."

Q: "Do you think they are used?"

*A: "I don't think so." Senior Doctor, Malaysia, Male*.

### The requirements for guidelines

Participants provided strong direction about what they needed in guidelines.

#### Evidence-based

All the participants were very clear that guidelines must be based on the best available research evidence if they are to be useful. They felt strongly that evidence should be used to ensure the guidelines are accurate and reliable, not only to ensure best practice but also to provide support in case of legal action.

*"Well if they're not based on evidence, who is saying it's the right way to do it?. If all the evidence says you should do it this way and this hospital decides to do it that way, from a medico-legal perspective it can't be very good." Senior Nurse, Australia, Female*.

In all hospitals, participants noted that most locally-developed guidelines were not developed in a rigorously evidence-based manner.

*"In house [guidelines are] just handed down from colleague to colleague and not evidence-based necessarily, but just current practice and what works and maybe what other hospitals do locally, but nothing really technically evidence-based." Allied Health Clinician, Australia, Female*.

#### Concise and clinically focused

The key message about the format of guidelines was that they should be clear, well-written, short and provide a quick answer to clinical decision of interest. Participants often suggested that a logical, step-by-step progression was important and that algorithms or flowcharts were useful.

Many Australian participants were deeply frustrated by guidelines in which the clinically relevant information was preceded by several pages of what they perceived as less important information about organisational requirements or administrative processes. Clinicians wanted the key clinical recommendations to be presented at the front of the guideline.

*"I find that often the information that you want is towards the end of it and it can be very long-winded and stuff that you don't need and ... especially if you're in emergency ... and you really need to find out something, it's not that easy to find." Junior Nurse, Australia, Female*.

Two very different thoughts were expressed by interviewees about the ideal format of guidelines: that they must be simple and quick to read and that they should be detailed and thorough. Many participants felt that a flowchart or algorithm alone was not enough, and that these should be followed by more detailed information explaining the reasoning behind the recommendations of the guideline and providing links to the original papers from which the evidence was drawn.

*"I quite like structures where the guideline exists in several layers. There's a very simple layer which is your day to day decision making where you don't necessarily even want to understand what underpins it. But you can dig deeper and deeper as you wish to do, particularly if you're faced with a point in the guideline that you're struggling to accept that it does actually apply to the clinical situation where you actually really do go back and say, 'okay, well what was the evidence at that point?"' Senior Doctor, Australia, Male*.

#### Credible

Participants wanted credible guidelines, with recommendations that were trustworthy. Assessment of the credibility of a guideline was based on a number of factors including respect for the developing organisation, approval of the guideline by the health service and clarity of the development methods, including clear searches for research evidence, broad consultation and piloting of the guideline.

*"I think it probably gets back to the process of how the guideline was formulated, so if it has been consultative, if there's been some evidence to support it or not support it. And then if it is trialled for a certain period of time." Allied Health Clinician, Australia, Female*.

#### Multidisciplinary

In Australia there was variation between senior and junior participants about whether guideline development should involve clinicians from across disciplines. All senior doctors and nurses, and most allied health professionals felt that it was essential that guideline development should be multidisciplinary, should involve both junior and senior staff, and that there should be wide consultation. Some junior doctors and nurses agreed, however several were surprised that multidisciplinary guideline development would be undertaken and were unclear about the value of input from clinicians from other disciplines.

*"I imagine that the nurses and physiotherapists and audiologists, they do have their guidelines. I can't see why a medical person would have to have too much input into development of that." Junior Doctor, Australia, Male*.

In most of the hospitals in South East Asia, having a multidisciplinary group responsible for guideline development, as is recommended by evidence-based guideline development methods, was not usual practice, nor was it explicitly valued. Most often the guidelines were largely developed by the senior medical staff.

Q: "Are nurses involved in writing the protocols?"

*A: "No. Consultants. Consultants and the specialists they are the ones who go in a closed room I think. ... Then they come up with the protocol." Senior Nurse, Malaysia, Female*.

However in some hospitals this was not the case, or was beginning to change and involvement of nurses and midwives in some aspects of the guideline development process was felt to be valuable.

*"Actually when we make the guidelines we invite the midwives to give their opinion, when like we're brainstorming..., the midwives give their questions too. And it's very important..." Senior Doctor, Indonesia, Female*.

Most participants in both Australia and South East Asia were unconvinced that consumer involvement in guideline development was important. They felt that while involving consumers in guideline development was potentially useful in some situations, it was not essential, particularly in technical areas, and it could create problems if consumers had difficulty understanding the concepts or could not remove themselves from their personal experience, so as to provide broader input.

*"To be honest I struggle to see where the value is, but I'd be willing to be convinced." Senior Doctor, Australia, Male*.

#### Up-to-date

Participants from all settings were very clear that guidelines need to be kept up-to-date. They suggested that guidelines should have expiry dates and that there should be a process of regular review when searches for additional evidence were undertaken. Several suggested that this review should be annual.

*"It would have to be a fluid document that could be changed as knowledge was gained. Because that is the problem with a lot of these things. They get done, but they don't get updated." Allied Health, Australia, Female*.

### The barriers to development of guidelines

The participants were very clear that there were some major barriers to development of guidelines to meet the needs they identified, and that as a result, these needs were not currently being met.

#### Lack of time

Unsurprisingly, the most frequently mentioned barrier to guideline development in all settings was time. Participants noted that rigorous guideline development takes many months and believed that neither they nor their staff or colleagues had the time to develop guidelines in methodologically rigorous ways. Participants emphasised that it was very difficult to quarantine or prioritise time for activities like guideline development when there were already enormous clinical pressures on their time.

*"There's just not enough time. We're busy enough as it is caring on the floor let alone being allotted the time to develop these guidelines." Senior Nurse, Australia, Female*.

In Australia, several participants suggested that one of the reasons for the time-consuming nature of guideline development was the process required to obtain organisational approval for guidelines, particularly when the committees involved met only infrequently.

The long time periods required to ensure broad consultation were another frequently mentioned reason for the substantial time required to develop guidelines.

#### Lack of skills

A lack of the skills required to develop guidelines in the local clinical environment was a commonly mentioned barrier. In Australia, participants perceived a lack of high-level skills in finding, appraising and interpreting research evidence, as well as in writing the guideline documents. Although several participants had experience in some of these aspects, few were confident that they or their colleagues had the level of these skills required to develop reliable evidence-based guidelines.

Q: "In this unit, does your team have the skills that they need to, if time wasn't an issue?"

*A: "Well the obvious answer to that is no." Senior Doctor, Australia, Male*.

Participants, and particularly nurses, in South East Asia frequently noted that lack of skill in English and lack of skill in using computers and the internet limited their ability to search for evidence

*"I do this ... search from website Cochrane Library but I have the problem with English, it's a little bit difficult for me to read." Junior Nurse, Indonesia, Female*.

#### Lack of access to evidence

As well as limited skills in finding evidence, participants also noted that for some clinical areas there was no evidence available on which to base guideline recommendations. In Australia, this was mentioned as a barrier to guideline development both in situations where evidence would be useful, but trials had not yet been conducted, and also in situations whose nature meant that trials would never be undertaken.

In South East Asia, interviewees were also limited in their ability to find and use research evidence because of limitations on availability of computers, internet access and full text journal articles.

*"We have internet access here, but our computer is non-functional right now. ... we're using only one for about 50 people actually." Junior Doctor, The Philippines, Female*.

#### Difficulty arranging meetings and achieving consensus

Another barrier common to all settings was that guideline development meetings with broad representation were hard to organise.

*"So if you want to meet with the four of us, it's almost impossible to have everybody free at one time. We can make a minimum of three, but ... it's so difficult actually, sometimes they have something on, and then we have something on, and it gets postponed. And it dies off." Senior Doctor, Malaysia, Male*.

The process of achieving consensus on the recommendations of the guideline was felt to be very important, especially in terms of enabling the implementation of the guideline, but also a potential barrier to development if agreement could not be reached.

A: "In our case, we are stopping the guidelines because there have been some problems ..."

Q: "So you've had to stop for a while, is it for clinical reasons? Because you were too busy or?"

*A: "No, not the clinical reason [laughs]...I think it's more on conflicting ideas". Junior Doctor, The Philippines, Female*.

There was also a concern among Australian participants that consensus processes were too easily dominated by more confident group members. Participants were concerned that nurses and more junior staff were less likely to feel comfortable to be assertive and contribute their perspectives, particularly if these were in conflict with views expressed by others. This was tempered by suggestions that they might become more comfortable if they had more experience in these situations.

#### The role of adaptation

Participants also talked about adapting guidelines from other hospitals or organisations for use in their own settings. While participants from all settings were generally positive about this approach, there were different levels of acceptance, with several participants suggesting that they would look at other guidelines to get background information or to confirm that local practice was in line with that recommended elsewhere, but that they didn't feel it was appropriate to use them directly.

Participants from all countries were concerned about both the relevance of externally developed guidelines to their clinical setting and also the lack of high-quality guidelines.

## Discussion

This research highlights that while guidelines are valued, there are major barriers to developing EBCPGs in hospitals. These include lack of time, skills, information technology (IT) infrastructure and access to research. These barriers reflect and build on those identified by other researchers.

Lack of time and skills have also been identified as barriers to EBCPG development by national professional bodies [17-19]. It has been suggested that the substantial time and skills required to undertake systematic reviews for EBCPG development have led some professional groups to revert to consensus-based CPG development [19]. Of particular importance to EBCPG development in hospital contexts is that skills in systematic reviewing of research literature are not commonly part of the training of clinicians and these skills are unlikely to be available to hospitals who seek to develop EBCPGs [20,21].

Previous research has established that IT resources are a basic requirement for finding and using research and that these are often not available in resource-poor settings [22,23]. The importance of access to research, in the form of subscriptions to journals, research databases and similar resources, to enable improvement in health care delivery has similarly been recognised in other studies [23,24].

Awareness of these barriers to developing EBCPGs has led to a call for more feasible methods of developing guidelines that balance rigour and pragmatism, and for research to examine *"the trade-off between rigor and pragmatism that currently challenges the guideline movement." *[19] Some authors have suggested that EBCPG development should be undertaken by methodologists rather than by clinical experts [25]. This approach would address two of the barriers identified in this study; clinicians would not have to be experts in systematic reviewing, nor find time above their clinical load to develop EBCPGs. However, development of EBCPGs led by methodologists would rely on availability of methodologists and funding, and this is unlikely to be achieved in hospitals.

This study has some limitations. The relatively small number of hospitals included limits the extent to which the results can be applied to other settings. However the hospitals were very diverse, including both rural and metropolitan environments in a high-income country and a variety of low and middle income countries. The consistency of the results reported across these hospitals suggests that the results may be applicable beyond the settings in which the research was conducted.

Similarly, the interviews were all undertaken in maternal and neonatal healthcare settings and so the results may not be generalisable to other clinical areas. However the substantial gap between research and practice and the resulting rates of preventable maternal and child morbidity and mortality, particularly in Asia and the Pacific where these studies were undertaken, make maternal and neonatal healthcare a priority for research [26].

Further research is required to determine the extent to which the results of this study reflect the value placed on, and experience of, EBCPG development in other contexts and clinical areas. Research is also required to develop pragmatic EBCPG development methods that are feasible in hospital settings and which produce guidelines which are trustworthy.

## Conclusions

The clinicians in these eleven very different hospitals all value guidelines and want EBCPGs that provide rapid access to reliable, up-to-date information to help them make the best possible decisions. However they feel that established EBCPG development processes that are enormously time, skill and resource intensive, are not feasible in their settings. They feel strongly that a more feasible way of developing guidelines, or of getting evidence into practice, is needed.

Most of the barriers to evidence-based guideline development identified by these participants were shared across all hospitals, however there were a few that were specific to particular contexts. The fact that the great majority of the identified barriers were shared across settings may provide an opportunity to develop a more pragmatic way of developing guidelines that can be applied in many contexts, with awareness of the specific additional barriers that are relevant in particular settings.

## Competing interests

The author declares that they have no competing interests.

## Authors' contributions

TT conceived of the project, developed the methods, undertook the interviews and the analysis, prepared the first draft of the article, revised it, read and approved the final manuscript.

## Pre-publication history

The pre-publication history for this paper can be accessed here:

http://www.biomedcentral.com/1472-6963/9/235/prepub

## Supplementary Material

Additional file 1 **Table 1**. Table displaying the indicative questions used in the study. Click here for file

Additional file 2 **Table 2**. A list of key points from the study. Click here for file
