# Supplementary material for: Developing evidence-based clinical practice guidelines in hospitals in Australia, Indonesia, Malaysia, the Philippines and Thailand: values, requirements and barriers
Source: BMC Health Serv Res. 2009 Dec 15;9:235. doi: 10.1186/1472-6963-9-235 (PMC2800111; doi:10.1186/1472-6963-9-235)
Supplement: Additional file 1 — Table 1. Table displaying the indicative questions used in the study. [file 1472-6963-9-235-S1.DOC]

| Table 1. Indicative Questions Can you describe the process for developing guidelines at your hospital?   - Who decides what topics the guideline will be developed for? - Who are the guidelines used by – doctors or nurses or both? - Who writes the guidelines? - Who reviews drafts of the guidelines? - Do they look for existing guidelines? If yes, where? - Do they look for research evidence? If yes, where? - Do they appraise the evidence/guidelines? If yes, how? - Are references to research included in the guidelines? - Is a summary of the research included in the guidelines? - How long does it take to write a guideline? - What do finished guidelines look like?   What are the barriers to development of evidence-based guidelines?   - Why is research evidence not used in guideline development? - Do you have access to research evidence? If yes, from where? - Do the people writing the guidelines - have training in how to do it? - know how to find research? - know how to find existing guidelines? - know how to evaluate research? - know how to evaluate existing guidelines? - have enough time to develop guidelines? - work with clinicians from other disciplines? Why/why not? - involve consumers?   What methods of guideline development might be useful in the future?   - What makes a guideline useful? Format, content, credibility, currency? - How could we make it possible for people to - look for existing guidelines? - look for research evidence? - appraise existing guidelines/research evidence? - include research in guidelines? - work together on guidelines with clinicians from other disciplines? - work on guidelines with consumers? - How long should it take to write a guideline? - What should finished guidelines look like? |
| --- |
