# Supplementary material for: Developing evidence-based clinical practice guidelines in hospitals in Australia, Indonesia, Malaysia, the Philippines and Thailand: values, requirements and barriers
Source: BMC Health Serv Res. 2009 Dec 15;9:235. doi: 10.1186/1472-6963-9-235 (PMC2800111; doi:10.1186/1472-6963-9-235)
Supplement: Additional file 2 — Table 2. A list of key points from the study. [file 1472-6963-9-235-S2.DOC]

## Table 2. Key Points

- The clinicians interviewed in this study, from very different countries and settings, want guidelines, and they want them to be evidence based
- They believe guidelines should
  - be developed by, and be designed for use by, clinicians
  - provide key clinical recommendations at the front of the document
  - provide detailed information on the evidence and reasoning behind the recommendations on following pages
  - be developed with broad consultation
  - be updated regularly
- Barriers to achieving this that were shared across countries include
  - lack of dedicated time
  - lack of skills in finding, appraising and interpreting evidence
  - difficulty arranging meetings and achieving consensus
  - difficulty with adapting existing guidelines
- Barriers to achieving this that were primarily identified in Australian hospitals include
  - cumbersome organisational guideline approval processes
  - a feeling that guidelines are being developed for bureaucratic ends
- Barriers to achieving this that were primarily identified in South East Asian hospitals include
  - difficulty accessing evidence due to limited resources available for computers, internet and journal subscriptions
  - difficulty accessing evidence due to limited skills in computing and English
